# Insulin resistance and dysglycemia are associated with left ventricular remodeling after myocardial infarction in non-diabetic patients

**DOI:** 10.1186/s12933-019-0904-3

**Published:** 2019-08-07

**Authors:** Chen Die Yang, Ying Shen, Lin Lu, Feng Hua Ding, Zhen Kun Yang, Rui Yan Zhang, Wei Feng Shen, Wei Jin, Xiao Qun Wang

**Affiliations:** 1 Department of Cardiology, Ruijin Hospital, Shanghai Jiao-Tong University School of Medicine, 197 Ruijin Road II, Shanghai, 200025 People’s Republic of China; 2 Institute of Cardiovascular Disease, Shanghai Jiao-Tong University School of Medicine, Shanghai, People’s Republic of China

**Keywords:** Insulin resistance, Dysglycemia, Left ventricular dilation, Remodeling, Myocardial infarction

## Abstract

**Background:**

Adverse cardiac remodeling after ST-segment elevation myocardial infarction (STEMI) is a major cause for poor cardiovascular outcomes such as heart failure. The predisposing factors and underlying mechanisms remain not fully understood. This study investigates the association of insulin resistance and dysglycemia with left ventricular (LV) remodeling after STEMI in non-diabetic patients.

**Methods:**

A total of 485 non-diabetic subjects with STEMI who underwent primary percutaneous coronary intervention were consecutively enrolled and followed up for 12 months. Relation of homeostasis model assessment-estimated insulin resistance (HOMA-IR) and glucose levels to changes in echocardiography parameters was studied.

**Results:**

Left ventricular dilation was detected in 49.1% of subjects at 12-month follow-up after STEMI, and was more severe in subjects with impaired fasting glucose (IFG), impaired glucose tolerance (IGT) and high HOMA-IR levels. HOMA-IR remained correlated to changes in LV dimensions after adjusting for confounding risk factors. Multivariate regression analysis demonstrated that higher HOMA-IR was independently associated with greater LV dilation after STEMI. A significant interaction term was present between HOMA-IR and IGT in the model (*P *= 0.001).

**Conclusions:**

Our study reveals that insulin resistance and dysglycemia are prevalent in non-diabetic patients with STEMI and are predictors of the post-infarction LV dilation.

*Trial registration* Trials number, NCT02089360; registered on March 17, 2014

**Electronic supplementary material:**

The online version of this article (10.1186/s12933-019-0904-3) contains supplementary material, which is available to authorized users.

## Background

Despite rapid advances in acute treatment and secondary prevention measures in recent years, the incidence of chronic heart failure (CHF) after myocardial infarction remains relatively high, and is associated with late mortality [1]. From the onset of myocardial infarction, left ventricular (LV) remodeling occurs in response to abrupt increase in ventricular loading and acute inflammation [2]. Multiple mechanisms are implicated in this process to compensate for the loss of cardiomyocytes [3, 4]. Maladaptive LV remodeling, especially in conditions of disturbed metabolism and neurohormone overaction, predisposes adverse cardiovascular outcomes [5–7].

Insulin resistance is a well-established composite index of systemic inflammatory and metabolic disorders [8]. Mounting evidence reveals that insulin resistance predicts and, to some extent, mediates the development of atherosclerosis [9], myocardial infarction [10] and in-stent restenosis [11]. The predictive role of insulin resistance for LV remodeling and incident CHF has also been proposed [12, 13]. Especially, a unique concentric LV remodeling pattern was characterized in relatively healthy subjects with insulin resistance [12]. However, the role of insulin resistance in LV remodeling after acute myocardial infarction remains poorly understood.

To investigate the relationship between insulin resistance and post-infarction LV remodeling, we analyzed non-diabetic subjects with acute ST-elevation myocardial infarction (STEMI) who underwent primary percutaneous coronary intervention (PCI) and performed echocardiography at baseline and 12-month follow-up. The associations between changes in LV geometric parameters and homeostasis model assessment-estimated insulin resistance (HOMA-IR) as well as glucometabolic disorders were examined.

## Methods

### Study population

This study complies with the Declaration of Helsinki. The study protocol was approved by the local hospital ethics committee, and written informed consent was obtained from all participants.

**Fig. 1:**
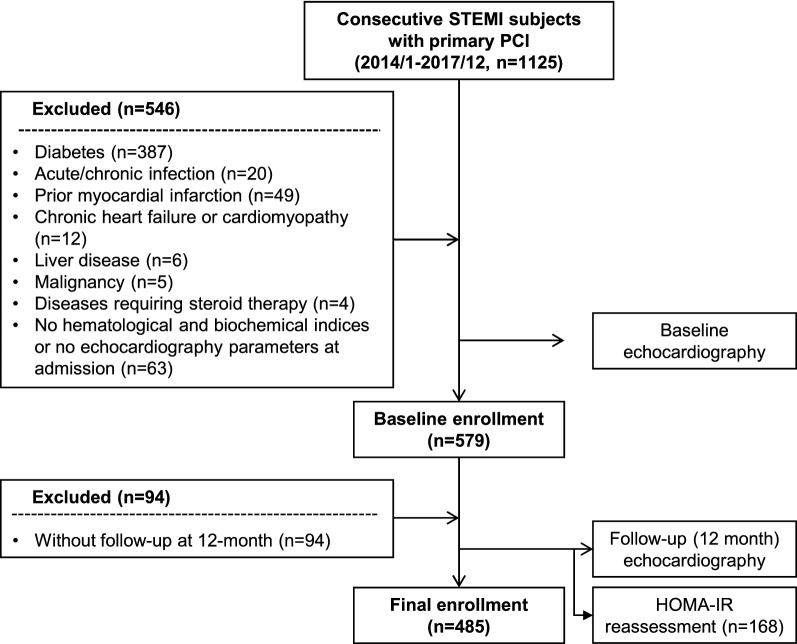
Flow chart of patient enrollment. *STEMI* ST-segment elevation myocardial infarction, *PCI* percutaneous coronary intervention

We consecutively enrolled 1125 subjects with the first ever acute STEMI with the onset of symptoms within 12 h preceding hospital admission and received primary PCI from Jan, 2014 to Dec, 2017 in the Department of Cardiology, Rui Jin Hospital, Shanghai Jiao Tong University School of Medicine (Fig. 1). A total of 483 patients comorbid with diabetes, chronic or acute infection, prior myocardial infarction, chronic heart failure or cardiomyopathy, liver disease, malignancy and diseases requiring steroid therapy, were excluded. Additionally, 63 patients who did not have biochemical indices including fasting glucose and insulin, as well as echocardiography parameters obtained at discharge were not enrolled. Another 94 patients without follow-up echocardiography at 12-month were also excluded. Thus, 485 patients comprised the final enrollment. HOMA-IR levels were reassessed in 168 subjects within the study population at follow-up. The changes in echocardiography parameters were calculated. The association between changes in echocardiograph parameters and basal HOMA-IR as well as glucometabolic disorders was analyzed. The diagnosis of diabetes was made according to the criteria of American Diabetes Association and prediabetes was defined by fasting blood glucose of 100 mg to < 126 mg/dL, 2-h plasma glucose of 140 to < 200 mg/dL, or HbA1C of 5.7 to < 6.5% [14]. Hypertension and dyslipidemia were diagnosed according to seventh report of the Joint National Committee on prevention, detection, evaluation, and treatment of high blood pressure (JNC 7) and guideline of the National Cholesterol Education Program (ATP III), respectively [15, 16].

### Clinical, biochemical and echocardiographic assessments

All the blood samples were drawn and echocardiography was performed at discharge except that the peak cardiac troponin I (cTnI) was recorded after admission. The blood samples were collected in a quiet, air-conditioned room after overnight fasting and after at least 20 min supine rest. Serum glucose, insulin, blood urea nitrogen, creatinine, uric acid, total cholesterol, low-density lipoprotein-cholesterol (LDL-C), high-density lipoprotein cholesterol (HDL-C), triglycerides, apolipoprotein A-I and apolipoprotein B were assessed (HITACHI 912 Analyzer, Roche Diagnostics, Germany). The estimated glomerular filtration rate (eGFR) was computed using the Chronic Kidney Disease Epidemiology Collaboration equation [17]. Blood HbA1c concentration was measured using ion-exchange high performance liquid chromatography with Bio-rad Variant Hemoglobin Testing System (Bio-Rad Laboratories, USA). Serum N-terminal pro-B-type natriuretic peptide (NT-proBNP) was determined using a commercially available electrochemiluminescence immunoassay kit (Roche Diagnostics). Serum levels of high sensitive C-reactive protein (hsCRP) were determined by ELISA (Biocheck Laboratories, Toledo, OH, USA). HOMA-IR was calculated according to the formula: fasting insulin (µU/L) × fasting glucose (mmol/L)/22.5. The detailed information about medical history and lifestyles including smoking status was obtained using a standard questionnaire by trained physicians. Body mass index (BMI) was calculated using the formula of weight/height^2^ (kilograms per square meter). Body surface area (BSA) was calculated using the formula of 0.0061 × height + 0.0128 × weight − 0.1529. Blood pressure was measured on the non-dominant arm in a seated position after a 10-min rest, using an electronic blood pressure monitor (OMRON Model HEM-752 FUZZY’ Omron Co., Dalian, China). Three measurements were taken at 1-min intervals, and the average was used for analysis.

Transthoracic echocardiography was performed, at least, at the time of enrollment and 12-month follow-up, using a commercially available system (Vivid-I, GE Healthcare, Milwaukee, WI) with a 1.9- to 3.8-mHz phased-array transducer. Echocardiography was performed by a single sonographer credentialed in cardiac ultrasound. Two-dimensional (2D), pulsed-Doppler imaging was performed from standard parasternal and apical transducer positions with 2D frame rates of 60 to 100 frames/s. All data were stored digitally, and offline data analysis was performed (EchoPac, version 7; GE Healthcare) by two cardiologists at the conclusion of the study, blinded to the study time point.

The LV ejection fraction (LVEF) was calculated using the modified Simpson’s biplane technique. The LV length was measured in the apical 4-chamber view. To facilitate application of clinical normality cut points (20), LV end-diastolic volume (LVEDV) and LV end-systolic volume (LVESV) were indexed by BSA calculated at each study time point. LV mass was estimated from M-mode measurements by the formula:

$$ {\text{LV mass }} = 0.8 \times 1.04 \times \left[ {\left( {LVEDD + IVST + LVPWT} \right)^{3} - LVEDD^{3} } \right] + 0.6, $$

where LVEDD is LV end-diastolic diameter, IVST is interventricular septal thickness, LVPWT is LV posterior wall thickness. LV mass was indexed by BSA. Relative wall thickness (RWT) was determined by the formula:

$$ RWT = \left( {IVST + LVPWT} \right)/LVEDD, $$

where IVST is interventricular septal thickness, LVPWT is LV posterior wall thickness, LVEDD is LV end-diastolic diameter.

### Statistics

Continuous variables were presented as median (interquartile range) or mean (standard deviation), and categorical data were summarized as frequencies (percentages). For continuous variables, normal distribution was evaluated with Kolmogorov–Smirnov test. Differences among groups were analyzed by Student’s t-test or one-way analysis of variance (ANOVA) followed by post hoc Bonferroni test. Correlation between HOMA-IR and changes in echocardiography parameters was determined by Pearson’s correlation test. Furthermore, multivariate linear regression was implemented to interrogate the association between insulin resistance and LV remodeling parameters. In model 1, changes in LVEDD were employed as dependent variables, and covariates, including male gender, age, history of hypertension, the presence of chronic kidney disease (CKD), levels of lymphocytes, HDL cholesterol, TIMI flow grade after PCI, log-transformed cTnI and baseline LVEDD was adjusted. In model 2, we further adjusted the presence of impaired glucose tolerance (IGT) and BMI (dichotomized by median), as well as interaction terms of each maker with tertiles of HOMA-IR in the model. Regression coefficients of interaction term represent the difference in coefficient of IGT (or BMI) between subgroups stratified by tertiles of HOMA-IR. Coefficients of interaction terms corresponding to intermediate and high tertiles vs. low tertile of HOMA-IR were calculated. All statistical analyses were performed using the SPSS 23.0 for Windows (SPSS, Inc., Chicago, IL, USA). A 2-tailed < 0.05 was considered statistically significant.

## Results

### Basic characteristics of the studied population

A total of 485 non-diabetic patients with STEMI who underwent PCI were enrolled in this study (Table 1). The level of HOMA-IR (2.36 [1.56–3.92]) was higher than the normal range according to previous reports [10, 18] and the prevalence of impaired fasting glucose (IFG) and IGT was 40.0% and 40.2%, respectively. After dividing the study population into three groups according to HOMA-IR tertiles, we found subjects with high HOMA-IR levels tended to have higher BMI, diastolic blood pressure, liver enzymes, triglyceride and cTnI levels, but were younger and had lower NT-proBNP levels. There was no difference in renal function, smoking history, comorbidities such as hypertension, atrial fibrillation, and CKD between the three groups. On admission, dual antiplatelet agents, statins, ACEI/ARBs and β-blockers were prescribed unless contraindicated. A total of 8.7% of patients were prescribed spirolactone depending on clinical scenario. No significant difference in medication use was detected between different groups.

**Table 1:** Baseline characteristics

HOMA-IR tertiles	Low	Intermediate	High	*P*-value
n	174	150	161	
Demographic characteristics				
Age, years, mean (SD)	65.24 (10.45)	62.37 (11.23)	59.93 (11.84)	< 0.001
Male, n (%)	154 (88.51)	132 (88.00)	149 (92.55)	0.342
Clinical measures, mean (SD)				
BMI, kg/m^2^	23.09 (3.00)	24.76 (2.93)	25.95 (3.78)	< 0.001
SBP, mmHg	121.07 (17.36)	124.71 (17.87)	125.37 (20.30)	0.078
DBP, mmHg	71.99 (11.45)	75.57 (11.98)	77.76 (12.02)	< 0.001
Current or former smoker, n (%)	87 (50.00)	72 (48.00)	66 (40.99)	0.228
Diseased vessels, n (%)				
Single-vessel disease	59 (33.91)	46 (30.67)	52 (32.30)	0.768
Multi-vessel disease	115 (66.09)	104 (69.33)	109 (67.70)	
Medical history, n (%)				
Hypertension	90 (51.72)	86 (57.33)	101 (62.73)	0.126
Atrial fibrillation or flutter	6 (3.45)	8 (5.33)	8 (4.97)	0.682
Chronic kidney disease	8 (4.60)	10 (6.67)	13 (8.07)	0.424
Cerebrovascular disease	20 (11.49)	8 (5.33)	16 (9.94)	0.140
Laboratory values, median (IQR) or mean (SD)				
HbA1c, %	5.60 (5.40–5.90)	5.75 (5.40–6.10)	5.70 (5.40–5.90)	0.116
Fasting glucose, mmol/L	5.04 (0.87)	5.45 (0.76)	6.17 (0.94)	< 0.001
Fasting insulin, μU/mL	5.61 (2.12)	10.32 (1.95)	21.67 (10.83)	< 0.001
Triglyceride, mmol/L	1.31 (0.99–1.73)	1.51 (1.08–2.07)	1.65 (1.18–2.30)	< 0.001
Total cholesterol, mmol/L	4.50 (1.44)	4.37 (0.83)	4.77 (1.06)	0.040
HDL cholesterol, mmol/L	1.04 (0.26)	1.02 (0.22)	1.02 (0.23)	0.855
LDL cholesterol, mmol/L	2.85 (1.31)	2.67 (0.68)	3.02 (0.86)	0.044
Apolipoprotein A–I, g/L	1.10 (0.20)	1.11 (0.19)	1.14 (0.18)	0.381
Apolipoprotein B, g/L	0.89 (0.29)	0.90 (0.21)	0.94 (0.19)	0.084
Alanine aminotransferase, IU/L	40.96 (23.55)	37.12 (18.47)	61.98 (44.55)	< 0.001
Aspatate aminotransferase, IU/L	140.90 (141.13)	137.10 (123.68)	238.58 (213.27)	< 0.001
Blood urea nitrogen, mmol/L	5.20 (4.10–6.20)	5.00 (4.20–6.00)	5.10 (4.20–6.50)	0.497
Serum creatinine, μmol/L	78.00 (62.25–87.00)	77.00 (67.00–87.00)	80.00 (68.00–92.00)	0.166
eGFR, mL/min/1.73 m^2^	83.42 (20.70)	88.11 (21.65)	85.38 (19.15)	0.178
hsCRP, mg/L	5.84 (2.31–13.74)	4.50 (2.06–11.47)	3.96 (1.81–12.85)	0.151
NT-proBNP, pg/mL	1049.00 (718.10–2668.00)	859.55 (309.30–1843.00)	466.60 (278.70–1344.75)	< 0.001
cTnI (ng/mL)	6.19 (0.35–29.86)	9.94 (0.32–36.47)	24.40 (1.78–75.45)	< 0.001
Medication use, n (%)				
ACEI or ARBs	125 (71.84)	114 (76.00)	133 (82.61)	0.064
Beta-blockers	146 (83.91)	122 (81.33)	135 (83.85)	0.787
Statins	170 (97.70)	142 (94.67)	157 (97.52)	0.243
Spirolactone	10 (5.75)	12 (8.00)	20 (12.42)	0.089

*ACEI* angiotensin-converting enzyme inhibitor, *ARB* angiotensin receptor blocker, *cTnI* cardiac troponin I, *eGFR* estimated glomerular filtration rate, *BMI* body mass index, *DBP* diastolic blood pressure, *HbA1c* glycated hemoglobin A1c, *HDL* high-density lipoprotein, *HOMA-IR* homeostatic model assessment-insulin resistance, *hsCRP* high-sensitivity C-reactive protein, *IQR* interquartile range, *LDL* low-density lipoprotein, *NT-proBNP* N-terminal pro-B-type natriuretic peptide, *SBP* systolic blood pressure, *SD* standard deviation

HOMA-IR was reassessed in 168 subjects within the study population at the 12-month follow-up. There was no statistic difference in baseline HOMA-IR levels between the selected subjects and the overall population (*P *= 0.122). Although HOMA-IR levels were lower at follow-up (2.12 [1.61–3.09]) compared to those in the baseline (2.61 [1.77–3.99]; *P *= 0.022), there was still a stepwise increase in the follow-up HOMA-IR levels across increasing tertiles of the baseline values (*P *< 0.001; Additional file 1: Figure S1).

### Changes in LV geometric and functional properties

**Fig. 2:**
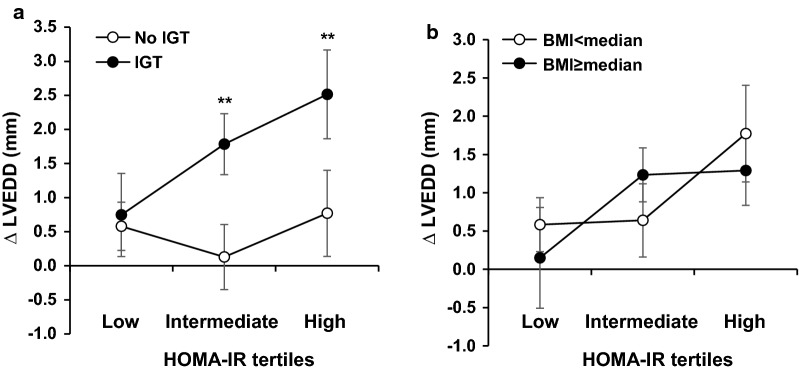
Distribution of changes in LVEDD among HOMA-IR tertiles stratified by IGT and BMI. Shown are distribution of changes in LVEDD according to HOMA-IR tertiles stratified by the presence of IGT (**a**) or dichotomized BMI (**b**) levels. Data are expressed as mean ± 95% confidence interval. *BMI* body mass index, *IGT* impaired glucose tolerance, *HOMA-IR* homeostatic model assessment-insulin resistance, *LVEDD* left ventricular end-diastolic diameter. **P < 0.01 vs. subjects without IGT within the same HOMA-IR tertile

Baseline and 12-month follow-up LV geometric and functional parameters were assessed. Changes in echocardiography parameters were compared in subjects stratified by HOMA-IR tertiles (Table 2). There was an upward trend in post-infarction LV dilation with increasing tertiles of HOMA-IR (LVEDD, LVESD, LVEDV index and LVESV index, all *P *< 0.001). Especially, subjects with IGT presented greater LV dilation than those without at intermediate and high tertiles of HOMA-IR (Fig. 2; intermediate tertile: 1.78 vs. 0.13, *P *= 0.001; high tertile: 2.52 vs. 0.77, *P *= 0.010). When stratified by BMI, however, no difference was detected at any tertile. On the other hand, the post-infarction LV wall thinning appeared to be more prominent (Δ IVST, *P *= 0.003; Δ LVPWT, *P* = 0.006; Δ RWT, *P *= 0.001) in subjects with high HOMA-IR, suggesting a trend toward eccentric remodeling of LV after STEMI in subjects with insulin resistance. No significant difference in changes in LV mass index (*P *= 0.130) and LVEF recovery (*P *= 0.089) during follow-up was detected between different tertiles.

**Table 2:** Changes in echocardiography parameters during follow-up grouped by HOMA-IR tertiles

HOMA-IR tertiles	Low	Intermediate	High	*P*-value
LVEDD, mm				
B	50.09 (4.35)	50.77 (5.00)	51.60 (5.23)	< 0.001
F	50.60 (4.99)	51.68 (5.49)	53.04 (5.33)	
Δ	0.52 (2.47)	0.91 (3.08)	1.44 (3.96)	
LVESD, mm				
B	34.04 (5.09)	34.93 (5.35)	35.55 (5.40)	< 0.001
F	33.89 (5.43)	34.93 (6.14)	36.60 (6.37)	
Δ	− 0.15 (3.351)	0.00 (2.94)	1.05 (4.11)	
LVEDVI, mL/m^2^				
B	70.17 (12.65)	69.61 (13.88)	70.28 (16.67)	< 0.001
F	72.09 (14.60)	72.66 (16.56)	75.00 (17.84)	
Δ	1.91 (7.96)	3.06 (10.63)	4.66 (13.20)	
LVESVI, mL/m^2^				
B	29.11 (9.97)	29.46 (10.55)	30.32 (11.55)	< 0.001
F	28.65 (11.00)	29.86 (12.95)	32.28 (14.21)	
Δ	− 0.46 (6.68)	0.40 (6.38)	1.96 (9.13)	
LVMI, g/m^2^				
B	93.55 (17.15)	96.39 (21.13)	96.21 (21.67)	0.130
F	94.47 (18.35)	95.32 (16.69)	93.66 (17.95)	
Δ	0.91 (15.84)	− 1.07 (17.75)	− 2.73 (15.24)	
IVST, mm				
B	9.27 (1.04)	9.55 (1.15)	9.60 (1.10)	0.003
F	9.14 (1.11)	9.36 (1.04)	9.01 (1.16)	
Δ	− 0.13 (1.21)	− 0.19 (1.22)	− 0.59 (1.48)	
LVPWT, mm				
B	8.92 (0.92)	9.20 (1.07)	9.19 (0.97)	0.006
F	8.93 (0.91)	8.92 (0.85)	8.83 (0.75)	
Δ	0.01 (1.03)	− 0.28 (1.09)	− 0.37 (1.25)	
RWT				
B	0.36 (0.04)	0.37 (0.05)	0.37 (0.05)	0.001
F	0.36 (0.04)	0.36 (0.05)	0.34 (0.05)	
Δ	− 0.01 (0.05)	− 0.01 (0.04)	− 0.03 (0.06)	
LVEF, %				
B	59.04 (8.03)	58.20 (7.85)	57.76 (7.35)	0.089
F	60.89 (7.63)	59.99 (7.53)	58.22 (8.36)	
Δ	1.85 (6.76)	1.79 (5.40)	0.47 (6.80)	

Values are given as mean (standard deviation)

*B* baseline, *Δ* changes in corresponding parameters, *F* follow-up, *IVST* interventricular septal thickness, *LVEDD* left ventricular end-diastolic diameter, *LVEDVI* left ventricular end-diastolic volume indexed to body surface area, *LVEF* left ventricular ejection fraction, *LVESD* left ventricular end-systolic diameter, *LVESVI* left ventricular end-systolic volume indexed to body surface area, *LVMI* left ventricular mass indexed to body surface area, *LVPWT* left ventricular posterior wall thickness, *RWT* relative wall thickness

In addition, when grouping the overall study population based on the presence of IFG or IGT, we found subjects with IFG or IGT present more severe LV dilation than those without these glucometabolic abnormalities (Additional file 1: Tables S1, S2). Dividing subjects according to prediabetes status showed a modest but non-significant increase in LVEDD (*P *= 0.051) and LVEDV index (*P *= 0.091) in subjects with prediabetes versus those with normoglycemia (Additional file 1: Table S3).

### Relation of insulin resistance parameters to LV dilation

**Fig. 3:**
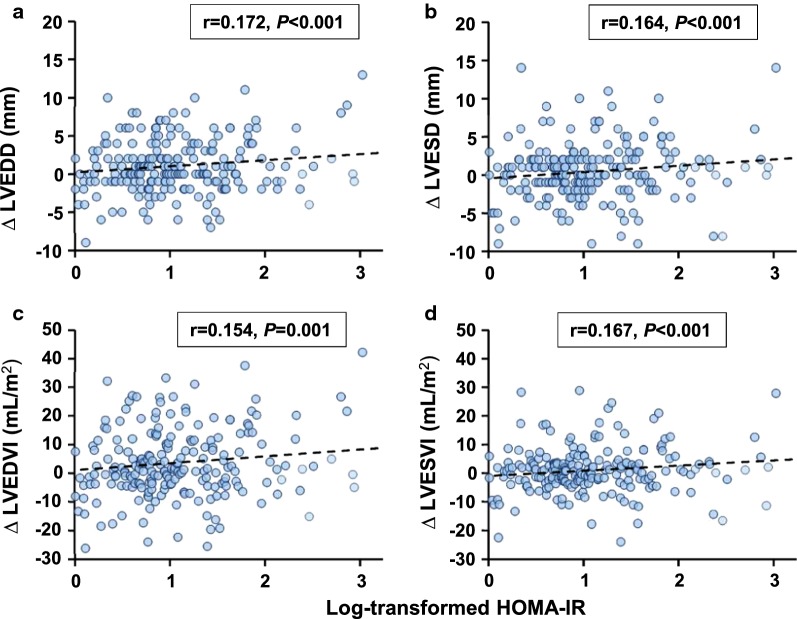
Correlation between Log-transformed HOMA-IR and changes in LV dimension. HOMA-IR was logarithmically transformed before plotting. *HOMA-IR* homeostatic model assessment-insulin resistance, *LVEDD* left ventricular end-diastolic diameter, *LVEDVI* left ventricular end-diastolic volume indexed to body surface area, *LVESD* left ventricular end-systolic diameter, *LVESVI* left ventricular end-systolic volume indexed to body surface area

Correlation analyses showed that Log-transformed HOMA-IR was correlated positively with changes in LV diameter and volume (LVEDD: r = 0.172, *P *< 0.001; LVESD: r = 0.164, *P *< 0.001; LVEDV index: r = 0.154, *P *= 0.001; LVESV index: r = 0.167, *P *< 0.001; Table 3 and Fig. 3), and inversely with LV wall thickness (IVST: r = − 0.129, *P *= 0.004; LVPWT: r = − 0.144, *P *= 0.001; RWT r = − 0.193, *P *< 0.001). After adjusting for confounding risk factors, these correlations remained significant. In line with previous reports [19], an inverse correlation was also present between Log-transformed HOMA-IR and changes in LVEF.

**Table 3:** Correlation analysis of Log-transformed HOMA-IR and ∆ echocardiography parameters

∆ Geometric/functional parameters	Log-transformed HOMA-IR			
	Unadjusted		Adjusted^a^	
	r	*P*-value	r	*P*-value
LVEDD	0.172	< 0.001	0.149	0.001
LVESD	0.164	< 0.001	0.163	< 0.001
LVEDVI	0.154	0.001	0.129	0.005
LVESVI	0.167	< 0.001	0.171	< 0.001
LVMI	− 0.047	0.303	− 0.106	0.022
IVST	− 0.129	0.004	− 0.152	0.001
LVPWT	− 0.144	0.001	− 0.203	< 0.001
RWT	− 0.193	< 0.001	− 0.219	< 0.001
LVEF	− 0.092	0.043	− 0.126	0.006

*IVST* interventricular septal thickness, *LVEDD* left ventricular end-diastolic diameter, *LVEDVI* left ventricular end-diastolic volume indexed to body surface area, *LVEF* left ventricular ejection fraction, *LVESD* left ventricular end-systolic diameter, *LVESVI* left ventricular end-systolic volume indexed to body surface area, *LVMI* left ventricular mass indexed to body surface area, *LVPWT* left ventricular posterior wall thickness, *RWT* relative wall thickness

^a^Adjusted for age, sex, history of smoking, hypertension and chronic kidney disease, and LVEF at baseline

### Multivariate linear regression analysis for HOMA-IR and LV dilation

Finally, we performed multivariate linear regression to interrogate the association between insulin resistance parameters and LV remodeling (Table 4). After adjusting for confounding clinical variables in Model 1, tertiles of HOMA-IR were independently associated with changes in LVEDD (*P* for trend < 0.001). Compared with the low tertile, intermediate and high tertiles of HOMA-IR corresponded to 1.196-mm and 1.926-mm increases in LVEDD, respectively. By including IGT, BMI (dichotomized by median) and the interaction terms of these variables with tertiles of HOMA-IR in Model 2, we found the presence of IGT (*P *= 0.007), but not BMI (*P *= 0.140), was independently associated with changes in LVEDD. Furthermore, IGT modified the association of HOMA-IR with changes in LVEDD (*P *= 0.001), suggesting that subjects with high HOMA-IR levels perceive greater risk of LV dilation when coinciding with IGT. In contrast, the interaction term between HOMA-IR and BMI was non-significant (*P *= 0.212), suggesting that HOMA-IR was associated with post-infarction LV dilation irrespective of BMI.

**Table 4:** Multivariate linear regression analysis for ∆LVEDD after STEMI

Covariates	Model 1			Model 2		
	Coefficient (95% CI)	Sβ	*P*-value	Coefficient (95% CI)	Sβ	*P*-value
Male gender	− 0.009 (− 1.044 to 1.025)	− 0.001	0.986	0.028 (− 1.004 to 1.061)	0.003	0.957
Age	− 0.190 (− 0.453 to 0.073)	− 0.066	0.157	− 0.125 (− 0.385 to 0.135)	− 0.043	0.345
Smoking	0.446 (− 0.138 to 1.030)	0.067	0.134	0.366 (− 0.218 to 0.949)	0.055	0.219
Hypertension	0.351 (− 0.220 to 0.923)	0.052	0.228	0.339 (− 0.226 to 0.905)	0.050	0.239
CKD	0.886 (− 0.220 to 1.991)	0.066	0.116	0.855 (− 0.230 to 1.939)	0.063	0.122
Lymphocytes	− 1.028 (− 1.563 to − 0.492)	− 0.166	< 0.001	− 1.049 (− 1.581 to − 0.517)	− 0.169	< 0.001
HDL cholesterol	− 3.118 (− 4.722 to − 1.515)	− 0.163	< 0.001	− 3.265 (− 4.878 to − 1.651)	− 0.170	< 0.001
TIMI flow ≤ 2	1.215 (− 0.460 to 2.890)	0.059	0.155	1.489 (− 0.182 to 3.161)	0.072	0.081
Log cTnI	0.109 (0.007 to 0.211)	0.092	0.036	0.114 (0.012 to 0.216)	0.096	0.029
Basal LVEDD	− 0.293 (− 0.359 to − 0.227)	− 0.435	< 0.001	− 0.317 (− 0.384 to − 0.251)	− 0.471	< 0.001
Basal LVEF	− 0.145 (− 0.187 to − 0.103)	− 0.344	< 0.001	− 0.145 (− 0.186 to − 0.104)	− 0.343	< 0.001
HOMA-IR			< 0.001*			0.088*
Tertile 2 vs. 1	1.196 (0.541 to 1.851)	0.170	< 0.001	− 0.361 (− 1.360 to 0.638)	− 0.051	0.478
Tertile 3 vs. 1	1.926 (1.237 to 2.614)	0.272	< 0.001	1.224 (0.095 to 2.353)	0.173	0.034
IGT	–	–	–	− 1.337 (− 2.311 to − 0.363)	− 0.185	0.007
IGT × HOMA-IR	–	–	–			0.001^†^
β_int_ (tertile 2 vs. 1)	–	–	–	2.453 (1.162 to 3.744)	0.260	< 0.001
β_int_ (tertile 3 vs. 1)	–	–	–	1.925 (0.561 to 3.288)	0.197	0.006
BMI	–	–	–	0.760 (− 0.249 to 1.769)	0.113	0.140
BMI × HOMA-IR	–	–	–			0.212^†^
β_int_ (tertile 2 vs. 1)	–	–	–	0.775 (− 0.566 to 2.117)	0.088	0.257
β_int_ (tertile 3 vs. 1)	–	–	–	− 0.405 (− 1.796 to 0.985)	− 0.049	0.567

*BMI* body mass index, *cTnI* cardiac troponin I, *CKD* chronic kidney disease, *HOMA-IR* homeostasis model assessment-estimated insulin resistance, *IGT* impaired glucose tolerance, *LVEDD* left ventricular end-diastolic diameter, *LVEF* left ventricular ejection fraction, *Sβ* standardized coefficient, *β*_*int*_ regression coefficient (interaction)

*P for trend; ^†^*P* for interaction

## Discussion

The major finding of the present study is that in non-diabetic patients with STEMI, the development of LV dilation is more severe in those presenting with insulin resistance characterized by high HOMA-IR levels and the presence of IFG or IGT.

### Insulin resistance predicts LV dilation

Insulin resistance serves as the pathophysiological basis of type 2 diabetes, as well as the primary metabolic disorders in a great number of patients long before progression into overt diabetes [20]. While diabetes is an established risk factor for the development of heart failure after myocardial infarction [21, 22], the role of insulin resistance in post-infarction LV remodeling in non-diabetic patients is less understood. Existing evidence showed that insulin resistance predicted subsequent CHF incidence independently of established risk factors including diabetes in a large community-based cohort [23]. A separate study of the same cohort revealed that insulin resistance was associated with LV concentric remodeling rather than LV hypertrophy [12]. However, the remodeling process triggered by acute myocardial infarction may be different from that occurred in chronic conditions.

In the present study, we demonstrated that LV dilation was more prominent in subjects presenting with insulin resistance and dysglycemia. There was an upward trend in LV dilation with increasing tertiles of baseline HOMA-IR levels in non-diabetic patients with STEMI. HOMA-IR was positively correlated to changes in LV dimensions. By constructing multivariate regression models, we found HOMA-IR was independently associated with LV dilation after STEMI. On the other hand, the post-infarction LV dilation was more severe in subjects with IFG or IGT. These data are consistent with previous reports that basal glucose levels and poor glycemic control are predictors of adverse LV remodeling and cardiovascular events [24–27]. Noteworthy, glycemic variability after the onset of acute coronary events was shown to predict patient prognosis and subsequent LV dilation [28, 29]. Taken together, these findings provide evidence that patients with insulin resistance, dysglycemia and high glycemic variability perceive higher risk of developing adverse LV remodeling and poor cardiovascular outcomes.

We found the association between LV dimension and HOMA-IR level was modified by the presence of IGT in the regression model. Subjects in the intermediate and high tertiles of HOMA-IR have greater LV dilation when coinciding with IGT, implying that insulin resistance and dysglycemia may have additive effects on adverse LV remodeling after myocardial infarction. In contrast, no significant interaction term was present between HOMA-IR and BMI with changes in LV dimension. This may be due to the reason that BMI is a crude measure of general adiposity and its impact on LV remodeling is, at least partly, attributable to its association with insulin resistance (Pearson r = 0.344, *P *< 0.001). In other words, subjects with different BMI but identical HOMA-IR level may develop LV dilation to a similar extent (Fig. 2b). A substantial body of evidence showed that abdominal obesity would be more strongly related to risk of mortality [30], myocardial infarction [31] and type 2 diabetes [32]. Although we did not record waist circumstance or waist-to-hip ratio in this study, they are expected to provide more information than BMI in relation to LV remodeling.

### Prevalence of insulin resistance among STEMI patients

The mean HOMA-IR level in this study is beyond the normal range as defined by a substantial number of epidemiological studies [10, 18]. There was also high prevalence of IFG (40.0%), IGT (40.2%) and prediabetes (72.6%) in the study population even though subjects who met the diagnosis of diabetes had already been excluded. These data concur with previous reports demonstrating high prevalence of glucometabolic abnormalities and undiagnosed diabetes in patients with acute myocardial infarction [33–35]. Although the observed hyperglycemia and compromised insulin action might be due to activated production of catecholamines and cortisol in response to infarct extension and myocardial dysfunction, Choi et al. [33] showed that the proportion of abnormal glucose tolerance kept similar at admission and 3 months after discharge when the effects of acute stress and inflammation should have already been lessened. In the present study, HOMA-IR levels were reassessed at 1-year follow-up in 168 subjects within the study population. Although there was a decrease in HOMA-IR at follow-up compared to the baseline, subjects with high HOMA-IR levels in the acute phase were still more likely to exhibit insulin resistance after 1 year.

### Possible mechanisms

We showed that subjects with insulin resistance were at higher risk to develop eccentric LV remodeling than those without after STEMI, which was different from previous findings [12] that insulin resistance led to concentric LV remodeling in chronic conditions. Different mechanisms may predominate in distinct pathophysiological conditions that may underlie the paradox. In the chronic phase, insulin resistance leads to altered substrate metabolism, maladaptive immune responses, mitochondrial dysfunction and endoplasmic reticulum stress. These mechanisms promote reactive oxygen species (ROS) production and inflammation, thereby resulting in interstitial collagen deposition, crosslinking, and finally myocardial hypertrophy and relaxation deficiency [36–38]. On the other hand, in the setting of myocardial infarction, insulin resistance is associated with poor myocardial reperfusion, impaired coronary microcirculation [39] and collateralization [40], and reduced collagen deposition in the scar [41]. These factors potentially lead to greater infarct size and post-infarction LV dilation, and finally a higher incidence of heart failure [42]. Nevertheless, the implicated mechanisms await precise characterization in future studies.

### Study limitations

We appreciate limitations in our study. First, this study was a retrospective analysis based on prospectively collected data, and all the enrolled patients were from a single center. Second, data were analyzed according to HOMA-IR levels near the acute phase, which may over-estimate insulin resistance status of STEM patients. Third, we evaluated LV remodeling by calculating changes in echocardiography parameters. Performing cardiac magnetic resonance would provide more information. Fourth, anthropometric parameters such as waist circumstance and waist-to-hip ratio were not recorded in this study. They are better measures of central obesity than BMI and should provide more information. Further prospective studies are warranted to analyze the causal link between insulin resistance and LV remodeling, and the prognostic value of HOMA-IR for hard cardiovascular events in subjects with STEMI.

## Conclusions

In conclusion, this study reveals high prevalence of insulin resistance and dysglycemia in non-diabetic patients with STEMI and their predictive value for subsequent adverse LV remodeling.

## Additional file

**Additional file 1: Table S1.** Comparison of Δ echocardiography parameters grouped by IFG dichotomy. **Table S2.** Comparison of Δ echocardiography parameters grouped by IGT dichotomy. **Table S3.** Comparison of Δ echocardiography parameters grouped by prediabetes status. **Figure S1.** HOMA-IR levels at follow-up grouped by HOMA-IR tertiles in the baseline.

## Data Availability

The datasets used and/or analyzed during the current study are available from the corresponding author on reasonable request.

## Abbreviations

CHF: chronic heart failure
LV: left ventricular
STEMI: ST-elevation myocardial infarction
PCI: percutaneous coronary intervention
HOMA-IR: homeostasis model assessment-estimated insulin resistance
cTnI: cardiac troponin I
LDL-C: low-density lipoprotein cholesterol
HDL-C: high-density lipoprotein cholesterol
eGFR: estimated glomerular filtration rate
NT-proBNP: N-terminal pro-B-type natriuretic peptide
hsCRP: high sensitive C-reactive protein
BMI: body mass index
BSA: body surface area
2D: two-dimensional
LVEF: LV ejection fraction
LVEDV: LV end-diastolic volume
LVESV: LV end-systolic volume
LVEDD: LV end-diastolic diameter
LVESD: LV end-systolic diameter
IVST: interventricular septal thickness
LVPWT: LV posterior wall thickness
RWT: relative wall thickness
ANOVA: one-way analysis of variance
CKD: chronic kidney disease
IGT: impaired glucose tolerance
IFG: impaired fasting glucose
ROS: reactive oxygen species
