# Supplementary material for: Insulin resistance and dysglycemia are associated with left ventricular remodeling after myocardial infarction in non-diabetic patients
Source: Cardiovasc Diabetol. 2019 Aug 7;18:100. doi: 10.1186/s12933-019-0904-3 (PMC6686425; doi:10.1186/s12933-019-0904-3)
Supplement: Supplementary file 1 — Additional file 1: Table S1. Comparison of Δ echocardiography parameters grouped by IFG dichotomy. Table S2. Comparison of Δ echocardiography parameters grouped by IGT dichotomy. Table S3. Comparison of Δ echocardiography parameters grouped by prediabetes status. Figure S1. HOMA-IR levels at follow-up grouped by HOMA-IR tertiles in the baseline. [file 12933_2019_904_MOESM1_ESM.pdf]

ONLINE SUPPLEMENTS

Table S1. Comparison of Δ echocardiography parameters grouped by IFG dichotomy.

ΔGeometric/functional parameters	Non-IFG (n=291)	IFG (n=194)	*P*-value
LVEDD, mm	0.42 (2.98)	1.74 (3.99)	<0.001
LVESD, mm	-0.11 (3.41)	0.91 (4.27)	0.004
LVEDVI, mL/m^2	1.39 (9.71)	5.92 (13.82)	<0.001
LVESVI, mL/m^2	-0.36 (6.98)	2.06 (9.53)	0.001
LVMI, g/m^2	-1.04 (15.86)	-0.66 (17.03)	0.805
IVST, mm	-0.23 (1.24)	-0.41 (1.43)	0.145
LVPWT, mm	-0.12 (1.09)	-0.34 (1.19)	0.038
RWT	-0.01 (0.05)	-0.02 (0.06)	0.002
LVEF, %	1.42 (6.24)	1.30 (6.65)	0.851

IFG, impaired fasting glucose; IVST, interventricular septal thickness; LVEDD, left ventricular end-diastolic diameter; LVEDVI, left ventricular end-diastolic volume indexed to body surface area; LVEF, left ventricular ejection fraction; LVESD, left ventricular end-systolic diameter; LVESVI, left ventricular end-systolic volume indexed to body surface area; LVMI, left ventricular mass indexed to body surface area; LVPWT, left ventricular posterior wall thickness; RWT, relative wall thickness.

Table S2. Comparison of Δ echocardiography parameters grouped by IGT dichotomy.

ΔGeometric/functional Parameters	Non-IGT (n=290)	IGT (n=195)	*P*-value
LVEDD, mm	0.60 (3.04)	1.46 (3.99)	0.007
LVESD, mm	0.16 (3.20)	0.50 (4.56)	0.337
LVEDVI, mL/m^2	2.07 (10.27)	4.80 (13.39)	0.012
LVESVI, mL/m^2	0.26 (6.84)	1.11 (9.81)	0.264
LVMI, g/m^2	-1.08 (15.54)	-0.59 (17.43)	0.748
IVST, mm	-0.26 (1.13)	-0.35 (1.57)	0.447
LVPWT, mm	-0.11 (1.06)	-0.34 (1.23)	0.026
RWT	-0.01 (0.05)	-0.02 (0.06)	0.013
LVEF, %	1.11 (5.74)	1.75 (7.27)	0.281

IGT, impaired glucose tolerance; IVST, interventricular septal thickness; LVEDD, left ventricular end-diastolic diameter; LVEDVI, left ventricular end-diastolic volume indexed to body surface area; LVEF, left ventricular ejection fraction; LVESD, left ventricular end-systolic diameter; LVESVI, left ventricular end-systolic volume indexed to body surface area; LVMI, left ventricular mass indexed to body surface area; LVPWT, left ventricular posterior wall thickness; RWT, relative wall thickness.

Table S3. Comparison of Δ echocardiography parameters grouped by prediabetes status.

ΔGeometric/functional Parameters	Normoglycemia (n=133)	Prediabetes (n=352)	*P*-value
LVEDD, mm	0.44 (2.80)	1.13 (3.69)	0.051
LVESD, mm	0.00 (3.07)	0.41 (4.05)	0.295
LVEDVI, mL/m^2	1.70 (9.14)	3.73 (12.50)	0.091
LVESVI, mL/m^2	-0.28 (5.58)	0.94 (8.94)	0.145
LVMI, g/m^2	-1.17 (15.69)	-0.78 (16.57)	0.816
IVST, mm	-0.23 (1.11)	-0.32 (1.40)	0.506
LVPWT, mm	-0.17 (1.13)	-0.22 (1.14)	0.710
RWT	-0.01 (0.05)	-0.02 (0.05)	0.268
LVEF, %	1.62 (5.58)	1.28 (6.69)	0.593

IGT, impaired glucose tolerance; IVST, interventricular septal thickness; LVEDD, left ventricular end-diastolic diameter; LVEDVI, left ventricular end-diastolic volume indexed to body surface area; LVEF, left ventricular ejection fraction; LVESD, left ventricular end-systolic diameter; LVESVI, left ventricular end-systolic volume indexed to body surface area; LVMI, left ventricular mass indexed to body surface area; LVPWT, left ventricular posterior wall thickness; RWT, relative wall thickness.

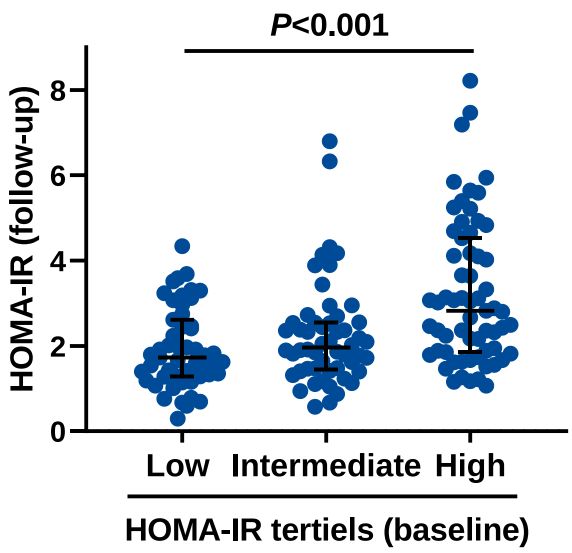

Figure S1. HOMA-IR levels at follow-up grouped by HOMA-IR tertiles in the baseline. HOMA-IR level was reassessed in 168 subjects within the study population. Shown are HOMA-IR levels at follow-up group by tertiles of their respective values in the baseline. Horizontal bars: low, 25 percentile; middle, median; upper, 75 percentile.
